# From Innovation to Validation: Strengthening Interventional Radiology Through Data

**DOI:** 10.1111/1754-9485.70109

**Published:** 2026-04-26

**Authors:** Kaiwen Cabbabe, Gerard S. Goh, Charles Li, Matthew Lukies

**Affiliations:** ^1^ Interventional Radiology Division, Department of Radiology Alfred Health Melbourne Victoria Australia; ^2^ Department of Surgery, School of Translational Medicine Monash University Melbourne Victoria Australia; ^3^ National Trauma Research Institute, School of Translational Medicine Monash University Melbourne Victoria Australia; ^4^ Department of Radiology Alfred Health Melbourne Victoria Australia; ^5^ Interventional Radiology Division, Imaging Department Monash Health Melbourne Victoria Australia

1

Interventional radiology (IR) is a modern patient‐facing clinical speciality, providing both procedural and longitudinal clinical care. The historic model of IR as an expert technical procedural‐only service is no longer fit‐for‐purpose in modern medicine. Innovation with new minimally invasive procedural techniques has been a cornerstone of IR practice and will continue. However, IR has relatively underperformed regarding the production of high‐quality clinical data to support practice. To provide the best patient‐centred procedural and clinical care, we must foster a culture of rigorous data‐driven practice.

Across Australia and New Zealand, physician‐ and surgical‐led specialities continue to dominate research output. This leadership is underpinned by structural advantages, such as competitive training pathways that reward academic output, established research units with dedicated personnel and infrastructure and funded non‐clinical time enabling clinicians to undertake research without compromising service delivery. Internationally, National Institutes of Health grant analyses from 2011 to 2020 confirm this, demonstrating that total research funding is overwhelmingly channelled towards physician disciplines, with surgical specialities ranking highly in the total number of grants per active clinician [1]. Such investment not only facilitates prospective studies but also incentivises sustained engagement in research, contributing to the development of influential clinical registries. A recent landmark Australian example includes the EXTEND‐IA TNK trial, which demonstrated Tenecteplase was superior to Alteplase for reperfusion in ischaemic stroke [2]. Another example is the ongoing Orthopaedic National Joint Replacement Registry, which continues to amass prospective data on arthroplasty patients to track surgical performance, revision rates and patient outcomes [3]. These examples illustrate the practice‐changing power of speciality‐driven, prospective data.

By contrast, IR remains a comparatively young speciality which is evolving rapidly, adopting increasing clinical responsibility, longitudinal patient care and complex decision‐making roles. Yet despite these advances and the speciality's progress towards formal recognition in Australia and New Zealand, prospective research output has lagged behind that of our more established physician and surgical colleagues. However, the appetite for research is evident in IR. A study by Clements et al. identified 34.7 manuscripts per 100 IR clinicians over a 6‐year period, reflecting strong academic interest and output [4]. However, most Australian IR research remains retrospective, limiting its capacity to influence policy and clinical practice.

Several barriers limit the cultivation of IR‐led research. First, as demand for IR services rises, procedural workload and service‐delivery pressures frequently displace research activity. This is often compounded by blended roles in which clinicians must balance procedural IR with diagnostic reporting, leaving limited protected time for prospective research. Second, IR in Australia and New Zealand is transitioning towards a clinician‐led speciality defined by consultation, ward‐based care, multidisciplinary decision‐making and longitudinal follow‐up [5]. However, speciality recognition is yet to be granted and fully accredited, and contemporary training pathways are still being implemented. Gaps in clinical practice privileges (e.g., admission rights, dedicated outpatient clinics and governance structures aligned to IR workflow) restrict longitudinal patient care and the ability to track outcomes, making prospective data collection challenging. These limitations, together with a comparatively small existing academic footprint, reduce IR's visibility and competitiveness for grant funding. Funding distribution and senior investigator recognition often favour specialities with established academic pathways and workforce structures. Third, trainees and early‐career IRs have struggled to find research mentors and structured academic training pathways that support research learning and output. Limited access to mentors and education impedes research skill acquisition, robust study design and sustained collaboration with other specialities. While these collective barriers exist in Australia and New Zealand, they are also seemingly evident within IR worldwide [6, 7].

An audit of abstracts presented at the Interventional Radiology Society of Australia (IRSA) annual scientific meetings in 2024 and 2025 reveals an imbalance in the distribution of retrospective and prospective studies. Although the total number of research presentations increased from 12 to 30 from 2024 to 2025, only 7 of the 42 abstracts presented prospective data [8, 9]. A similar pattern was seen at the Royal Australian and New Zealand College of Radiologists (RANZCR) scientific meetings, where 15 IR and Neuro‐IR oral abstracts were accepted in 2025, up from 11 in 2024 [10, 11], but only a single study was prospective. Moreover, a manual search of the Australian and New Zealand Clinical Trials Registry identified eight approved IR‐led prospective studies (Table 1) [12]. Only one was an approved registry [13]. These figures highlight the need to harness the enthusiasm for research among the IR community into the prospective datasets required to validate emerging IR techniques.

**TABLE 1:** List of registered prospective IR‐led trials identified on ANZCTR as of 8 December 2025.

Study title	Registry ID	Year registered	Study type	Procedure	Primary site(s)
Bearing nsPVA embolization for uterine artery embolization (BETTER‐UAE)	NCT06153667	2023	Prospective, multicentre observational study	Bearing nsPVA embolisation particles for uterine artery embolisation	Alfred Health (VIC, NSW)
Genicular artery embolisation vs. Sham procedure for knee pain secondary to total knee replacement.	ACTRN12623001218684	2023	Prospective, randomised double‐blind trial	Genicular artery embolisation	Royal Perth Hospital (WA)
A prospective study of the safety and effectiveness of uterine artery embolization for the treatment of endometriosis (UAE‐E) – a pilot study	ACTRN12622001301752	2022	Prospective pilot interventional study	Uterine artery embolisation	Alfred Health (VIC), Royal Women's Hospital (VIC)
Prospective registry for geniculate arterial embolisation as treatment for osteoarthritis and neovascularity.	ACTRN12622000621718	2022	Prospective IR registry	Genicular artery embolisation	Multi‐site (QLD)
A prospective, randomised, single‐blinded, sham controlled study of pelvic vein embolisation for treatment of erectile dysfunction.	ACTRN12620001023943	2020	Prospective, randomised, sham‐controlled trial	Pelvic vein embolisation	Alfred Health (VIC)
Instylla Hydrogel Embolic System Pilot Study for patients requiring transcatheter embolization of vascular bleeds	ACTRN12620000349943	2020	Prospective, non‐randomised, single‐arm study	Vascular embolisation	Alfred Health (VIC)
Prostate artery embolization for patients with lower urinary tract symptoms due to benign prostate hyperplasia	ACTRN12617001235392	2017	Prospective randomised controlled trial	Prostate artery embolisation	Christchurch (NZ)
Evaluating transcatheter arterial embolisation for improvement of pain in osteoarthritis (OA) of the knee	ACTRN12616001184460	2016	Prospective randomised controlled trial	Genicular artery embolisation	Barwon Health (VIC)

[Correction added on 28 May 2026, after first online publication: The sentence “This list excludes” has been removed from the table caption in this version.]

Without prospective data, innovation risks outpacing validation. Clinical trials and registries are not academic luxuries; they are mechanisms that define which treatments should be adopted, refined or abandoned. This has been demonstrated in IR‐led examples internationally. The COLLISION trial, a landmark randomised study comparing thermal ablation with surgical resection for small colorectal liver metastases, demonstrated non‐inferior overall survival, superior per‐tumour control and fewer complications with thermal ablation [14]. Its findings repositioned IR‐led ablation of hepatic colorectal cancer metastases from a palliative intervention to a curative‐intent alternative, which changed multidisciplinary referral pathways and redefined treatment algorithms. Conversely, the SWEDEPAD II trial showed that paclitaxel‐coated devices failed to deliver meaningful improvements in amputation‐free survival or long‐term outcomes in peripheral arterial disease [15]. This should prompt a reassessment of device selection in patients with intermittent claudication or non‐critical limb ischaemia. These studies demonstrate the power of prospective IR evidence to validate therapies and redirect practice when existing assumptions yield different results.

In this landscape, Interventional Radiologists and IRSA can play a leading role in the prospective data‐driven validation of minimally invasive procedural work. IRSA has recently championed the application to reinstate public funding for Vertebroplasty under the Medicare Benefits Schedule (MBS) item 35,401 [16]. This mandate requires practitioners billing the item to contribute procedural and outcome data to a national reporting mechanism, effectively establishing a real‐time registry. IRSA's biannual aggregation and reporting to the Department of Health ensures ongoing oversight, safety evaluation and transparent use of public funds.

Advancing IR‐led prospective research requires more than encouraging registry development; it necessitates building the structural and academic foundations that allow research to flourish. In recent years, the RANZCR has taken deliberate steps to embed research more firmly within the radiology training pathway, signalling a broader shift towards cultivating a culture of research. The RANZCR Research Action Plan outlines mechanisms to measure and strengthen research engagement, including incentivising trainees to undertake higher research degrees, improving access to formal biostatistics education and expanding the pool of competitive research funding [17]. Complementing these initiatives, the RANZCR Standards of Practice for Interventional Radiology and Interventional Neuroradiology now stipulate that service rosters should allocate protected research time, acknowledging that research is not ancillary to clinical practice but integral to it [18]. Integrating these initiatives and developing new research pathways with dedicated protected research time within the evolving IR programme will be essential to fostering this culture. However, policy alone will not deliver the level of evidence required for the continued advancement of IR, and a comprehensive research strategy is needed.

Establishing dedicated IR clinical academic positions affiliated with university medical programmes would support junior doctors and radiology trainees in pursuing research. Such positions provide a pragmatic mechanism for building mentorship capacity while encouraging academic teaching within IR units. They also facilitate collaboration with established research specialities and enable partnerships with other health services to develop multicentre research initiatives. Furthermore, creating clinician‐academic training pathways similar to the Academic Foundation Program or Academic Clinical Fellowship in the United Kingdom may encourage early trainees to pursue research and higher research degrees later in their careers [19, 20]. Equally important is the development of robust departmental infrastructure, including radiology research units staffed by research managers, clinical research fellows, data managers and research nurses. Such units provide the structure, methodological expertise and continuity necessary to transform concepts into robust, impactful studies. Access to simulation facilities, preclinical animal research platforms and active research laboratories further supports the full translational pathway from concept development to procedural innovation and, ultimately, to prospective clinical trials or registry formation. Additionally, collaboration with experts in computer science and engineering will be central to advancing device development and procedural navigation technologies for new techniques. Finally, collaboration with professional societies such as IRSA for grant funding, as well as partnerships with industry manufacturers to compete for first‐in‐human device trials, will be essential to provide more supportive opportunities in creating impactful studies. We summarise our overall strategic recommendations in Table 2.

**TABLE 2:** Suggested actions to strengthen IR‐led research in Australia and New Zealand.

Recommendation	Strategic action
1.	Formal recognition of IR as a speciality with a comprehensive model of care
2.	Development of a structured IR training programme incorporating protected research time and dedicated research opportunities
3.	Establishment of IR clinical academic positions within university‐affiliated medical programmes
4.	Greater engagement of radiology trainees in research during registrar training through RANZCR initiatives
5.	Development of radiology research units within major IR centres supported by dedicated research staff
6.	Creation of grant funding pathways through professional societies (e.g., IRSA) and third‐party device manufacturers
7.	Access to simulation and virtual reality laboratories, preclinical laboratories and animal research facilities
8.	Promotion of multisite and cross‐speciality collaborative research with IR

Cultivating a strong research culture remains a shared responsibility for the IR community. While our recommendations (Table 2) will require sustained commitment and incremental progress over time, maintaining momentum will be essential to establishing the foundations for high‐quality prospective research and robust clinical registries. Ultimately, strengthening this research ecosystem will ensure that the minimally invasive therapies we deliver are supported by rigorous, data‐driven processes.

## Author Contributions

**Kaiwen Cabbabe:** conceptualization, data curation, formal analysis, visualization, writing – original draft, methodology, project administration, writing – review and editing, software, resources. **Gerard S. Goh:** conceptualization, data curation, formal analysis, visualization, writing – original draft, methodology, project administration, writing – review and editing, software, resources. **Charles Li:** conceptualization, data curation, formal analysis, visualization, writing – original draft, methodology, project administration, writing – review and editing, software, resources. **Matthew Lukies:** conceptualization, data curation, formal analysis, visualization, writing – original draft, methodology, project administration, writing – review and editing, software, resources.

## Funding

The authors have nothing to report.

## Ethics Statement

The authors have nothing to report.

## Conflicts of Interest

Professor Gerard Goh is an Editorial Board member of JMIRO and co‐author of this article. He was excluded from editorial decision‐making related to the acceptance of this article for publication in the journal. The authors have no other conflicts of interest to declare.

## Data Availability

Data sharing not applicable to this article as no datasets were generated or analysed during the current study.
